# An Experimental Study of the Effects of Puncture of the Heart in Cases of Chloroform Narcosis

**Published:** 1887-11

**Authors:** B. A. Watson

**Affiliations:** Surgeon to Charity, St. Francis, and Christ Hospitals, Jersey City, New Jersey


### AN EXPERIMENTAL STUDY OF THE EFFECTS OF PUNCTURE OF THE HEART IN CASES OF CHLOROFORM NARCOSIS.

By B. A. Watson, A.M., M.D.,

SURGEON TO CHARITY, ST. FRANCIS, AND CHRIST HOSPITALS, JERSEY CITY, NEW JERSEY.

---

There has been, already, considerable written on cardicentesis, although the operation was originally proposed and performed in 1882, by Dr. B. F. Westbrook, of Brooklyn, N. Y. Prior to this date several unintentional punctures of the heart had been made, without being followed by unfavorable results.

Dr. John B. Roberts reported two cases of this kind at a stated meeting of the College of Physicians of Philadelphia, January 23, 1883. He likewise called attention to the fact that "Cloquet, Bouchut, Legros and Onimus have also observed the apparent innocuousness of wounds of the heart made with capillary trocars. Steiner found, ten years or more ago, that electro-puncture needles could be quite safely introduced into either ventricle, provided they were at once withdrawn."

Dr. F. M. Corwin reports in detail a case in which a puncture was made in the right ventricle, about one inch from its apex, without giving rise to any unfavorabe symptoms. Dr. C. L. Dana has reported two cases, in which punctures of the right ventricle were made with negative results. The reports in both these cases are so meagre as to diminish greatly the value which they would otherwise possess; but the comments on cardicentesis are unquestionably highly valuable.

Having referred briefly to the literature of cardicentesis, which possesses an important bearing on our subject, although we believe this operation has never been performed on the human subject for the relief of chloroform narcosis, we will now briefly state the observation which prompted us to make these experiments.

While working in my laboratory, with my assistant, on the evening of February 20, 1887, I killed, with chloroform, a bulldog, which weighed forty-one pounds; and about four minutes after the cessation of respiration made an incision from the base of the neck in the median line to the symphysis pubis. This incision was promptly carried completely through the walls of the thorax and abdomen—thus exposing the heart, lungs, intestines, etc. The heart was observed to be entirely motionless and in full diastole, while the lungs were completely collapsed and lying in the posterior portion of the thoracic cavity, in close proximity to the vertebral column. My assistant at this moment seized the heart between his thumb and fingers, when the cardiac action was immediately resumed, and the hand was removed.

The contractions promptly became full and regular, and during the first two minutes the pulsations were about sixty per minute, while during the succeeding minutes they became irregular and stopped at the end of this period, but only to be stimulated into action by the second touch—action continuing two minutes—and was the third time started up in the same manner, but this time continued to beat only one-half minute, and could not again be roused into action.

The lungs during this whole period remained completely motionless, and there were no other indications of life.

These observations suggested to me the possibility of arousing the heart into action, even after the entire cessation of its movements, by the introduction of a needle into this organ.

